# A Review of Foodborne Bacterial and Parasitic Zoonoses in Vietnam

**DOI:** 10.1007/s10393-013-0884-9

**Published:** 2013-10-26

**Authors:** Juan J. Carrique-Mas, J. E. Bryant

**Affiliations:** Wellcome Trust Major Overseas Programme, Oxford University Clinical Research Unit, Hospital for Tropical Diseases, 764 Vo Van Kiet, W.1, Dist.5, Ho Chi Minh City, Vietnam

**Keywords:** Vietnam, foodborne zoonoses, livestock, aquaculture, human–animal interface

## Abstract

Vietnam has experienced unprecedented economic and social development in recent years, and the livestock sector is undergoing significant transformations. Although food animal production is still dominated by small-scale ‘backyard’ enterprises with mixed crop–livestock or livestock–aquatic systems, there is a trend towards more intensive and vertically integrated operations. Changes in animal production, processing and distribution networks for meat and animal products, and the shift from wet markets to supermarkets will undoubtedly impact food safety risks in Vietnam in unforeseen and complex ways. Here, we review the available published literature on bacterial and parasitic foodborne zoonoses (FBZ) in Vietnam. We report on clinical disease burden and pathogen prevalence in animal reservoirs for a number of important FBZ, and outline opportunities for future research.

## Introduction

Foodborne zoonoses (FBZ) are human infections transmitted through ingested food and caused by pathogens whose natural reservoir is a vertebrate animal species (Hubalek 2003). In industrialized countries, ~20% people suffer annually from foodborne infections (Hall et al. 2005; Painter et al. 2013); the fraction attributable to zoonotic organisms is ~50% (Liu et al. 2004, 2006; Chen et al. 2010; EFSA 2012). In Vietnam, suspect outbreaks of foodborne disease are reported to the Vietnam Food Administration (VFA) (http://vfa.gov.vn). In 2011, 148 outbreaks were reported, with 38,915 cases, 3,663 hospitalizations and 27 deaths. In most cases, FBZ aetiologies remain undetermined, and the relative disease burden compared to other infectious diseases cannot be readily quantified.

Some characteristics of animal production and food consumption habits in Vietnam that may promote zoonotic disease transmission include: (1) high density of both human and animal populations living in close proximity; (2) a predominance of smallholder production systems with mixed species and little/no biosecurity; (3) the presence of abattoirs and wet markets operating with rudimentary hygiene, limited cold chain for distribution and low levels of meat inspection; (3) widespread consumption of raw/undercooked blood, meat, fish, organ tissues, raw leaf vegetables and wild animal products and (4) use of untreated wastewater and sewage for agriculture. For these reasons, Vietnam and South East Asia are often considered a hotspot for emerging infectious diseases (Coker et al. 2011). Indeed, the threat of emerging viral pathogens has received significant international attention, while the burden of endemic (predominantly bacterial and parasitic) zoonoses remains largely neglected. Within the last two decades, Vietnam has undergone extraordinary development. Changes underway involve rapid urbanization, intensification of animal production, modernization of food marketing systems and changes in food consumption habits. These changes will undoubtedly have major impacts on human exposures to animal pathogens, and hence the overall risk of zoonotic disease transmission. Despite significant investments in improved disease surveillance systems, information on FBZ is not readily available, and veterinary services are chronically under-resourced. The objectives of this review paper are to highlight knowledge gaps on FBZ and suggest priorities and specific areas for future research.

## Methods

We reviewed the available published literature in English from Vietnam on bacterial and parasitic FBZ from 1991 until January 2013. We searched PubMed for articles on food and waterborne zoonotic pathogens listed by the UK Health Protection Agency (HPA 2013), plus: ‘yersiniosis’ and ‘*Yersinia enterocolitica*’, ‘*Fasciola*’, ‘fascioliasis’, ‘*Angiostrongylus cantonensis*’, ‘fishborne trematodes’ and ‘*Paragonimus*’. Each search term was used in combination with ‘Vietnam’ and ‘Viet Nam’. We also used the same search terms to identify relevant articles published in the following Vietnamese public health and veterinary journals: (1) Tạp chì phòng  bệnh  rét và các bệnh ký sinh trùng (Journal of Prevention of Malaria and Parasitology); (2) Khoa học kỹ thuật thú y (Journal of Veterinary Medicine); (3) Y học thực hành (Medical Practice); (4) Y học tp. HCM (Medicine in Ho Chi Minh City); (5) Tạp chí Y học dự phòng (Journal of Preventive Medicine). None of these journals is electronically indexed. Although the emergence of antimicrobial resistance (AMR) is of paramount importance to food safety and public health in Vietnam (Dyar et al. 2012; Lestari et al. 2012), we have chosen not to address AMR, as this topic merits an extensive review on its own. Similarly, due to space limitations, we have not covered viral FBZ. We document available data on diverse FBZ, including human clinical impact and prevalence/incidence data within animal reservoirs, with a specific focus on the current situation in Vietnam.

## Bacterial FBZ

### Non-Typhoid Salmonella (NTS)

Non-typhoidal *Salmonella* (NTS) infections are caused by serovars of *Salmonella enterica* other than (non zoonotic) *S. typhi* or *S. Paratyphi*. Most NTS serovars are presumed to be zoonotic and potentially pathogenic to humans. NTS infections are typically self-resolving gastroenteritis, although complications may occur in children (<5 years), elderly and immunodeficient patients (Pegues and Miller 2010). NTS may infect a wide range of animals (both domestic and wild), but the vast majority do not to cause detectable pathology in the reservoir host.

In Vietnam, improvements in sanitation have resulted in dramatic reductions of typhoid over recent decades. In southern Vietnam, *S. typhi* cases reduced from 497 in 1994 to 34 in 2008, with a concurrent increase of invasive cases of NTS (from 9 to 24) (Nga et al. 2012). Studies on NTS in humans in Vietnam are summarized in Table 1. NTS prevalence in various farm animals (pre-slaughter) and in slaughter and retail facilities is summarized in Table 2. Detected levels in meat are high, suggesting widespread contamination during slaughtering/processing. Contaminated fish products likely reflect widespread use of animal/human sewage in aquaculture. Serovar or molecular data from animals and humans are limited, so it is difficult to establish the main sources of human infection. Epidemiological data suggests that person-to-person transmission plays a major role (Thompson et al. 2012). NTS carriage has been detected in ~5% of healthy adults (personal communication).

**Table 1:** Studies investigating the contributions of non-typhoidal *Salmonella* (NTS) to human diarrhoeal disease in Vietnam.

Citation	Study date	Study location	Sample size	Age	Study type	NTS prevalence	Other aetiologies and observations
Ngan et al. (1992)	1988–1989	Hanoi	83 diarrhoea cases	<3 years	Hospital-based study	No cases detected	24% had ETEC isolated, 8% had EPEC, 5% rotavirus, 6% *Candida*, and 4% *Giardia lamblia*
Isenbarger et al. (2001)	1998–1999	Red River Delta (3 communes)	1,655 healthy children in longitudinal study; 2,160 diarrhoea cases; 203 controls	<5 years	Longitudinal (community), hospital-based case-control study	0.8% cases, 1% controls	Diarrhoea incidence: 1.3 episodes/child/year. Only bacterial aetiologies investigated: Main aetiologies (cases vs. controls): *Campylobacter* spp. (6.8 vs. 3.8%), *Shigella* spp. (6.5 vs. 1.5%), ETEC (6.5 vs 4.4%)
Bodhidatta et al. (2007)	2001	Hanoi	291 diarrhoea cases; 291 controls	<5 years	Hospital-based case-control study	7% cases; 1% controls	Main aetiologies (cases vs. controls): Rotavirus (31% vs. 3%); Aeromonas (15% vs. 8%); Astrovirus (12% vs. 1%); *Shigella* (9% and 1%); *Campylobacter* (4% and 0%); Adenovirus (4% vs. 1%) and ETEC (3.0% vs. 0%)
Nguyen et al. (2004); Vu Nguyen et al. (2006)	2001–2002	Hanoi	587 diarrhoea cases; 249 controls	<5 years	Hospital-based case-control study	No cases detected	Main aetiologies (cases vs. controls): Rotavirus (46.7 vs. 3.6%), EAEC (11.6% vs. 7.2%), EPEC (6.6 vs. 4.4%), ETEC (2.2 vs. 0.4%); *Shigella* spp. (4.8 vs. 0%). *Campylobacter* isolation not attempted.
Khan et al. (2010)	2001–2003	International study including hospital in Hue (central Vietnam)	3611 febrile patients	5 to 15 years	Hospital-based	No cases detected	*S. typhi* detected in 18 cases (0.5%) in Hue; International study. Other sites in Pakistan, India, and Indonesia also included
Hien et al. (2007)	2002–2004	Hanoi; suburban area using wastewater for agriculture and aquaculture	222 children enrolled in longitudinal study; 111 diarrhoea cases; 111 controls	<6 years	Longitudinal (community), hospital-based case-control study	3.6% cases; 2.7% controls	Diarrhoea incidence: 0.63 episodes/child/year. Aetiologies (cases vs. controls): Rotavirus (17.1% vs. 4.5%), *Entamoeba hystolitica* (15.3% vs. 4.5%), diarrhoeagenic *E. coli* (22.5 vs. 23.4%), *Shigella* spp. (6.0 vs. 0%), *Campylobacter* spp. (1.8 vs. 1.8%)
Do et al. (2007)	2002–2004	Red River Delta	636 healthy adults in longitudinal study; 163 cases and 163 controls	15–70 years	Longitudinal (community), hospital-based case–control study	0.6% cases; 3.1% controls	Diarrhoea incidence: 0.28 episodes per adult per year. Aetiologies (cases vs. controls): *E. hystolitica* (9.9 vs. 0%); Diarrhoeagenic *E. coli* (13.5 vs. 9.8%); *Shigella* (3.1 vs. 1.2%); *C.* *jejuni* (0.6 vs. 0%); rotavirus (3.7 vs. 0.6%)
Thompson et al. (2012)	2009–2010	HCMC	1,419 diarrhoea cases	<5 years	Hospital-based study	5.4% cases (of which 58% were Group B)	Main independent risk factors: diarrhoeal contact (OR = 6.0) and living in a household with >2 children (OR = 2.3)

**Table 2:** Studies investigating NTS in food animals, meat and processed meat products of Vietnam.

Citation	Study date	Study location	Sampling site, species, sample type	Sample size	NTS prevalence; additional observations	NTS serovars
Tran et al. (2004)	2000	Mekong Delta (6 provinces)	Animals in farms: pigs (faeces), chickens and ducks (caecal samples)	439 pigs, 302 chickens, 357 ducks	Prevalence in pig, chicken and duck samples was 5.2, 7.9, and 8.7%, respectively. Higher prevalence on small-scale farms than industrial farms	Most common serovars: *S*. *Javiana* and *S. Derby* (pigs); *S. Emek* and *S. Javiana* (chickens); *S. typhimurium* and *S. Weltvreden* (ducks)
Vo et al. (2006)	2004	South Vietnam (13 provinces)	Pigs, cattle, chickens, ducks (carcasses, faeces, meat) at farms and abattoirs; Human (faeces)	Pigs (534), Cattle (390), Chickens (257), Ducks (34)	Prevalence in pigs, cattle, chicken and duck samples: 49.4, 27.4, 38.5, 20.5%, respectively.	Most common serovars: *S. typhimurium* and *S. Anatum* (pigs); *S. Emek* and *S. Blockley* (poultry); *S*. *Anatum*, *S. Weltevreden*, and *S. Lexington* (15.9%) (cattle)
Hong et al. (2006)	2004	Central Vietnam	Pigs on smallholder farms (faeces)	100 farms; 90 piglets with diarrhoea, 63 piglets without diarrhoea	No difference in prevalence of NTS in piglets with and without diarrhoea (10 and 11% positive, respectively)	
Phan et al. (2005)	2000–2001	Mekong Delta	Fresh meat and shrimps from the market	718 samples of meat (pork, duck, beef, chicken) and shrimps	70% (pork); 49% (beef); 24% (shrimps); duck (22%); 21% (chicken)	Most common serovars: *S. Derby*, *S. Weltrvreden*, and *S. London* (pork); *S*. *Weltevreden*, *S. London*, *S. Dessau* (beef); *S. Emek*, *S. typhimuirum*, *S. Dessau* (chicken); *S. Lexington*, *S. Derby*, and *S. Dessau* (duck); *S. Dessau*, *S. Weltvreden* and *S. Tennessee* (shrimps)
Van et al. (2007)	Unknown	HCMC	Fresh meat market samples	130 samples of meat	64% (pork); 62% (beef); 18% (chicken).	
Ha and Pham (2006)	2003–2004	Hanoi	Meat samples from factory, schools, hospital canteens	177 meat samples	8.3% poultry meat; 1.2% other meat	
Thai et al. (2012)	2007–2008	Northern Vietnam	Retail supermarkets	586 meat samples	39.6% (pork); 42.9% (chicken)	Most common serovars: *S. Emek*, *S. Infantis*, *S. Blockey*, and *S. Anatum* (chicken); *S. Anatum*, *S. Derby*, *S. typhimurium* and *S. Infantis* (pork)
Le Bas et al. (2006)	Unknown	Hanoi	15 pig slaughterpoints (faeces, carcass swabs)	117 faeces (caeca) and 46 carcass swabs	52% (faeces) and 96% (carcass swabs)	
Ellerbroek et al. (2010)	Unknown	Hanoi	6 pig slaughterpoints (lymph nodes)	178 lymph nodes	Prevalence from backyard small-scale farms (43%) versus intensive farms (29%)	*S. Derby* (50%); *S. typhimurium* (27%). Most *S. typhimurium* isolates were phage type DT22
Ta et al. (2012)	Unknown	Six provinces (different regions)	Wet markets and supermarkets (chicken carcasses)	1,000 carcasses	46%; no significant difference between study sites, temperature at retail, or wet markets versus supermarkets	

### Campylobacteriosis

Globally, *Campylobacter* is the single most common human bacterial diarrhoeal pathogen, and together with NTS, account for ~90% of foodborne bacterial disease. In Vietnam, as in other countries, *C. jejuni* is the dominant species found in paediatric clinical cases (~85%) (Isenbarger et al. 2001), with the remainder due to *C. coli*.

Reported *Campylobacter* prevalence in Vietnamese poultry meat ranges from 28 to 31% (Ha and Pham 2006; Luu et al. 2006). A 2005–2006 investigation of *Campylobacter* spp. at slaughterpoints in five cities worldwide indicated lowest prevalence in Ho Chi Minh City (HCMC) (15.3%, vs. an overall prevalence of 65.5%); 74% were *C. lari*, 9% *C.* *coli*, 4% *C.* *jejuni* and 13% other species. Semi-industrial poultry slaughtering was associated with lower contamination than informal direct slaughter by sellers (Garin et al. 2012). In Vietnam, there are no published data on pre-slaughter (on-farm) prevalence or *Campylobacter* species diversity.

The relative contribution of *Campylobacter* and NTS to diarrhoea is not particularly high, and asymptomatic infections appear to be common (Table 1). Given the widespread prevalence of NTS and *Campylobacter* in food products, and the intense human–animal exposures for most rural Vietnamese, the low incidence of clinical disease may reflect high levels of population immunity.

### Listeriosis

*Listeria monocytogenes* causes abortion and sepsis-like infection in humans, especially among immunocompromised individuals, neonates, pregnant women and the elderly. Clinical *L.* *monocytogenes* infection was confirmed in 2008–2009, among three patients with meningitis in Hanoi (Chau et al. 2010; Tran et al. 2010). Listeriosis has been linked to consumption of unpasteurised soft cheeses, processed meat and fish products. A study of fish and seafood products from Nha Trang Bay (central Vietnam) identified *L. monocytogenes* in 5.8% (Beleneva 2011).

There are no data on prevalence of *L. monocytogenes* in meat products in Vietnam, but studies in the region (Thailand) suggest a high prevalence of *L. monocytogenes* in raw meats, especially in those sold in supermarkets (Indrawattana et al. 2011). In Vietnam, meat is increasingly bought from supermarkets, especially in urban areas.

### *Streptococcus suis*

*Streptococcus suis* is an emerging human infection in Vietnam. The clinical picture is typically severe, and may involve skin, respiratory, neurological, cardiovascular and gastrointestinal systems. The largest *S. suis* outbreak recorded occurred in China in 2005, with 215 confirmed cases among pig slaughterers (Yu et al. 2006). Aetiological studies in Vietnam of cerebrospinal fluid from >2,000 patients (1996–2010) with suspect CNS infection have identified *S. suis* serotype 2 in 8.9–33.6% diagnosed patients (Mai et al. 2008; Wertheim et al. 2009b; Ho Dang Trung et al. 2012), confirming *S. suis* as the most frequent cause of bacterial meningitis in adults. About 66% patients experienced hearing loss as a sequela (Mai et al. 2008). Serotype 2 accounts for 96% of human cases, but other serotypes (i.e. 16, 14) have also occurred (Nghia et al. 2008). A case–control study identified the following risk factors: (1) eating undercooked pig blood/intestine; (2) occupation related to pigs; and (3) exposure to pigs while having skin injuries (Nghia et al. 2011). Due to poorly regulated marketing systems, ill pigs may enter the food chain, thus posing a significant risk to both slaughterhouse workers and consumers. Consumption of pig blood, intestines and organ meats is common in Vietnam (Wertheim et al. 2009a).

*Streptococcus suis* carriage rates of 41% (*n* = 542) have been identified in healthy Vietnamese pigs. Serotype 2 appears to be dominant (14%), followed by serotypes 3, 21, 21 and 16 (Ngo et al. 2011). High numbers of pigs infected with Porcine Respiratory Reproductive Syndrome (PRRS) virus have tested positive for *S. suis* in blood, indicating concurrent viraemic and bacteraemic infections (Hoa et al. 2013).

### Leptospirosis

Leptospirosis is caused by several pathogenic species within the genus *Leptospira*. Humans become infected through cuts, skin abrasions or by drinking contaminated water. Symptoms can range from mild, influenza-like illness to severe infection with renal and hepatic failure, pulmonary distress and death (Adler and de la Pena Moctezuma 2010).

Studies of acute jaundice in Hanoi and HCMC from 1993 to 1997 (*n* = 550 patients) reported 8 and 2% leptospirosis, respectively. The most commonly identified serovars were Seramanga and Bataviae (Laras et al. 2002). A serosurvey in the Mekong Delta reported high seropositivity (21%) among 36–45 year olds, with detection of Bataviae (21.7%), Panama (15.2%), Icterohaemorrhagiae (13.7%), and Australis (8.7%). In that study, walking barefoot was a significant risk factor for seropositivity, but not contact with animals (Van et al. 1998). A 2003 survey of children (*n* = 961) in southern Vietnam identified anti-*Leptospira* IgG in 12.8%, a 1.5:1 male: female ratio of seropositivity, and significant association with swimming in rivers. Based on IgG seroconversion, a 0.99% annual incidence was estimated (Thai et al. 2006).

Leptospiras have a broad range of animal reservoirs. Most studies in Vietnam have focused on pigs due to their impact on swine reproduction. In the Mekong Delta, Bratislava, Icterohaemorrhagiae, Automnalis, Grippotyphosa and Pomona are the most common serovars, with higher prevalence in small-scale farms compared to large holdings (Boqvist et al. 2002a, b). In general, there appears to be little overlap between serovars in pigs and humans; however, there is a paucity of surveillance data on which to judge exposures and epidemiological associations. The diffuse clinical picture and lack of straightforward diagnostics for leptospirosis (Wagenaar et al. 2004; Smythe et al. 2009) hamper adequate case reporting from Vietnam.

## Parasitic FBZ

### Toxoplasmosis

Toxoplasmosis is caused by the larval stage of the protozoan *Toxoplasma gondii*. Humans become infected by ingesting cysts (from undercooked meat/viscera), or oocysts released from the definitive host (the domestic cat) that contaminate food, water or the environment. Clinical signs range from mild to severe due to invasion of muscle, brain and eyes. Congenital toxoplasmosis occurs due to primary maternal infection during gestation (Montoya et al. 2010).

In Vietnam, a number of *T. gondii* serosurveys have been conducted (Table 3). Human seroprevalence is not particularly high (1–24%); in animals it ranges from low/medium (3% buffalo, 10% cattle) to high (23% pigs; 29% poultry; 50% domestic dogs). There are no published data on prevalence in domestic cats. Pigs are likely to play a major role in *T. gondii* infection, since pork is the most commonly consumed meat. In Thailand, a high prevalence in stray dogs has also been reported (Jittapalapong et al. 2007). Domestic dogs may also be relevant to transmission, since stray dogs are often imported from Thailand to supply dog meat restaurants. In southeast Asia, culinary habits (e.g. eating undercooked meat) and low water quality may be a more significant risk factor for *T. gondii* than cat ownership (Nissapatorn et al. 2003).

**Table 3:** Published surveys of *Toxoplasma gondii* in humans and domestic animal species in Vietnam.

Citation	Study date	Study location	Species	Details	Sample size	Overall prevalence; additional observations
Sery et al. (1988)	1984	Suburban Hanoi and Hoa Binh (northern mountain region)	Human	Healthy individuals, all ages	259 (140 from Hanoi, 119 from Hoa Binh)	24.3% (Hoa Binh); 15.7% (suburban Hanoi); Higher prevalence in early childhood than middle age.
Huong et al. (1998)	1995	Near HCMC	Cattle, buffalo	Cross-bred Frisian-Zebu cattle	200 of each species	10.5% (cattle) and 3% (buffalo)
Dubey et al. (2008)	2003	Mekong Delta (6 provinces) (and 6 other countries: Ghana, Indonesia, Poland and Italy	Chickens	From 38 different farms	330	24.2% seropositive by MAT
Huong and Dubey (2007)	2003–2005	Southern Vietnam (Dong Nai, Tien Giang provinces)	Pigs		587	27.2% seropositive in market weight pigs (6 months). Prevalence higher in older pigs
Dubey et al. (2007)	2006	Mekong Delta (7 provinces)	Domestic dogs		42	50% seropositive; experimental infections of naive cats with tissues from 8 dogs with high titres demonstrated transmission in 100% cases; high genetic similarity between *T.gondii* isolates from Vietnam and South America
Udonsom et al. (2008)	2007	Three provinces: Nghe An and Lao Cai (north) and Tien Giang (Mekong Delta)	Humans	Rural	650	Overall prevalence 4.2%; Highest in Nghe An (6.4%), followed by Lao Cai (4.7%) and Tien Giang (1.1%)

### Cryptosporidiosis

Cryptosporidiosis is caused by protozoa of the genus *Cryptosporidium*. Of ~20 *Cryptosporidium* species, seven are zoonotic (Fayer 2004), the most common one being *C. parvum* bovine genotype 2. Transmission is through ingested contaminated water and vegetables, although person-to-person transmission has been also documented. Most outbreaks have been attributed to *C. parvum* and linked to a waterborne source (Clinton White 2010). Studies in Vietnam have not found evidence of *Cryptosporidium* clinical disease among children with diarrhoea (Uga et al. 2005; Bodhidatta et al. 2007).

Cattle are thought to be the most common source of *C. parvum* genotype 2, although infection of pigs has also been described (Jenkins et al. 2010). A study of 266 cattle in three central provinces found 33.5% *C. parvum* positive (Nguyen et al. 2007a). Another study from the Red River Delta failed to detect *Cryptosporidium* among 68 healthy calves, but found 50% positive for *Giardia* (Geurden et al. 2008). A *Cryptosporidium* prevalence of 18% among diarrheic pigs of central Vietnam was reported (Nguyen et al. 2012), however, speciation was not performed, thus the implications for zoonotic transmission were unclear. *C.* *parvum* has been detected in farmed fish from southern Vietnam in association with wastewater used in aquaculture (Gibson-Kueh et al. 2011).

### Giardiasis

*Giardia lamblia* is a protozoan cause of diarrhoea found in soil, food, and water contaminated with faeces from infected humans or animals. *G. lamblia* has a very broad host range, and some subtypes/species are zoonotic. Recent molecular analysis of specific genetic assemblages suggests a high degree of host-specificity, with limited potential to infect humans (Xiao and Fayer 2008). A study on children less than 3 years old with severe diarrhoea in Hanoi identified *G.* *lamblia* among 2.4% (Ngan et al. 1992). Healthy people (*N* = 2,522) in north-western Vietnam had a surprisingly high prevalence (4.1%) (Verle et al. 2003). A study in calves less than 3 months old showed that *Giardia* spp. were the most prevalent parasites (50%); further characterization of 17 isolates indicated that all were non-zoonotic *G. duodenalis* (Geurden et al. 2008). Both *Giardia* and *Cryptosporidium* represent a challenge to safe drinking and recreational water supplies, due to their resistance to chlorine and environmental persistence.

### Taeniasis/Cysticercosis

Taeniasis and cysticercosis are distinct disease entities caused by different life stages of *Taenia* spp. Taeniasis refers to human enteric infection with the adult tapeworm, after ingestion of taenid cysts (*cysticerci*) present in undercooked beef (*T. saginata*) and pork (*T. solium* and *T. asiatica*). Cysticercosis are infections caused by ingestion of taenid eggs. Over the past decades, incidence of cysticercosis has decreased substantially worldwide owing to improved animal husbandry, sanitation and better meat inspection (Sotelo 2003).

Studies on taeniasis and cysticercosis in humans are shown in Table 4. During the 1990s, approximately 100–150 patients with neurocysticercosis were annually referred to Hanoi hospitals (Ky and Van Chap 2000). In addition, serosurveys published in Vietnamese suggest a large variation in prevalence among adults (0.2–7.2%) (Willingham et al. 2003).

**Table 4:** Published surveys of Taeniasis/Cysticercosis in humans in Vietnam.

Citation	Study date	Study location	Type of study	Details	Sample size	Overall prevalence; additional observations
Erhart et al. (2002)	1999	Bac Ninh (Red River Delta)	Survey using serum cysticercosis prevalence	Healthy individuals, all ages	210	5.7%; 5/12 seropositive individuals reported history of epilepsy.
Verle et al. (2003)	1999	Hoa Binh (north-western Vietnam)	Survey of gastrointestinal helminth infection	6 ethnic groups	526 households (2,522 samples)	Taenia eggs detected in 0.1% stool samples. One person had subcutaneous nodules that were diagnosed as cysticercosis by biopsy
Somers et al. (2007)	2002–2003	Northern Vietnam (14 provinces)	Hospital-based	Patients	65 patients from 14 hospitals	55.4% specimens identified as *T. asiatica*; 38.5% *T. saginata* and 6.2% *T. solium* tested by mitochondrial 12S rDNA by PCR
Somers et al. (2006)	2003–2004	Bac Kan (far northern province); Ha Tinh (central Vietnam); Hai Duong (Red River Delta)	Survey using serum (prevalence of cysticercosis) and faeces (prevalence of taeniasis)	Healthy individuals from 3 areas: 1. Bac Kan (rural, mountainous) 2. Ha Tinh (rural, coastal) 3. Hai Duong (peri-urban, costal)	303 (mountainous region); 179 (rural coastal region); 229 (peri-urban, coastal region)	Study investigating helminth infections. 5.3% (Bac Kan); 0.6% (Ha Tinh); 0% (Hai Duong)

Pig infections with *cysticerci* may result in reduced carcass value or full condemnation. A 1989–1992 study of meat carcasses in Hanoi indicated low prevalence (<0.1%). A 1999–2000 swine serosurvey indicated ~10% prevalence of *cysticerci*; however, cysts were *T. hydatigena*, for which the domestic dog is the final host (Dorny et al. 2004). Taenid eggs and *T. solium* cysts have been found in vegetables and dog meat sold in Hanoi (Uga et al. 2009; Willingham et al. 2010). Eating raw/pickled pork (i.e. ‘nem chua’) may be a major risk factor, as well as agricultural use of human wastewater as fertilizer (Dorny et al. 2004). To date, *T. asiatica* has not been reported from pigs in Vietnam, suggesting there may be other non-porcine intermediate hosts (Dorny et al. 2007). It is not yet clear whether *T. asiatica* causes cysticercosis (Galan-Puchades and Fuentes 2009). The presence of both *T.* *saginata and T.* *asiatica* in Vietnam may limit transmission of the more serious *T. solium* infection due to cross-protection (Conlan et al. 2009).

### Trichinellosis

Trichinellosis is caused by ingestion of encysted larvae of the genus *Trichinella*, predominantly from contaminated pork. *T. spiralis* is the most common species, found in pigs, wild boars and other species (Pozio et al. 2009). In humans, the clinical spectrum ranges from mild fever to myalgia and fulminating fatal disease. Like cysticercosis, the incidence of *Trichinellosis* has been decreasing worldwide over the last century. Data on *Trichinella* from Vietnam are limited to a few reports of sporadic outbreaks (~25 cases each) reported since 1970 in remote northern provinces (Dien Bien, Yen Bai and Son La), all traced back to consumption of undercooked/fermented pork (Taylor et al. 2009). A 2008–2009 serosurvey for *T. spiralis* in 1,035 free-roaming pigs reported age-dependent increases in seroprevalence, with overall seropositivity of 20%, and *Trichinella* larvae in 14.5% (Thi et al. 2010).

### Fascioliasis

Fascioliasis is caused by liver flukes of two species, *Fasciola hepatica* and *F. gigantica*. Humans become infected through ingestion of water or freshwater plants with adherent metacercaria (Mas-Coma 2005; Ashrafi et al. 2006) or juvenile forms (Taira et al. 1997). The parasite requires replication in *Lymnea* snails as intermediate hosts.

In Vietnam, fascioliasis has been increasingly diagnosed since the 1990s, mostly in ruminant-producing areas during the rainy season (De et al. 2003) (Table 5). Aberrant clinical forms (cutaneous fascioliasis) have been reported in association with *F. gigantica* (Xuan et al. 2005; Le et al. 2007). A hybrid of *F.* *hepatica* and *F.* *gigantica* has been reported from humans, cattle (Le et al. 2008) and goats (Nguyen et al. 2009). Parasite burdens are likely to have important economic repercussions for livestock production.

**Table 5:** Published surveys of *Fasciola* spp. in humans and ruminants in Vietnam.

Citation	Study date	Study location	Species	Sample collections	Sample size	Overall prevalence; additional observations
Tran et al. (2001a, b)	1997–2000	Hospitals in central and southern Vietnam	Humans	Stools from hospitalized patients with confirmed *Fasciola* infection	500	Largest number of cases from central provinces of Khanh Hoa, Binh Dinh and Quang Nga; prevalence per site per year unknown
Verle et al. (2003)	1999	Hoa Binh (north-western Vietnam)	Humans	Stools from healthy community cohorts (6 ethnic groups)	2,522 from 526 households	No *Fasciola* eggs detected
Holland et al. (2000)	1999–2000	Hanoi province (northern Vietnam)	Cattle	Faeces	119	22% *Fasciola* egg-positive; positives only among animals >3 months; no evidence of seasonality
Linh et al. (2003)	2000–2002	Hanoi province (northern Vietnam)	Cattle/buffalo	Faeces and livers	30 cattle, 2 water buffalo	62% *Fasciola* egg-positive, and 100% positive for worms in liver tissue
Anderson et al. (1999)	2002	Hanoi city (northern Vietnam)	Cattle	Faeces and livers	92	78.3% cattle had *Fasciola* in liver. Positive correlation between age of cattle and number of liver flukes
Suzuki et al. (2006)	2002–2003	Hanoi province (northern Vietnam)	Cattle	99 smallholder dairy farms; 4 time points; faeces	263 cattle	10% *Fasciola* egg-positive in June; 26% egg-positive in March; significant association between poor reproductive performance and *Fasciola* infestation
Uga et al. (2005)	2003–2004	Suburban Hanoi (northern Vietnam)	Humans	Stools from adolescents (14–15 years)	116	1% *Fasciola* egg-positive. The most frequently detected helminths were: *Trichuris trichiura* (67%), *Ascaris lumbricoides* (34%) and hookworm (3%)
Geurden et al. (2008)	2006	Red River Delta (5 provinces)	Cattle	Faeces	334 cattle	28% *Fasciola* egg-positive (3-24 moths); 39% prevalence in cattle >2 years
Nguyen et al. (2011)	2008	Binh Dinh (central Vietnam)	Cattle	Faeces and sera	825 cattle	54.9% *Fasciola* egg-positive and 72.2% *Fasciola* seropositive

It is unclear whether increasing case numbers of fascioliasis represent disease emergence or improved laboratory diagnostics and reporting. Changes in environmental factors and/or livestock production (i.e. increasing stocking densities, use of cattle faeces as fertilizer) may contribute to transmission (Tran et al. 2001b; De et al. 2003).

### Fish-Borne Zoonotic Trematodes (FZT)

FZT comprise a large group of flukes of the families *Heterophyidae*, *Echinostomatidae* and *Opistorchiidae* (Chai et al. 2005). Adult liver flukes live in the biliary tract of a range of vertebrates. Eggs are released in the environment; the miracidium penetrates freshwater snail tissues, where it develops into free-swimming cercariae that infect cyprinid freshwater fish. Within the fish host, parasites invade muscle and transform into metacercariae that are infectious for humans. Although most human FZT infections are subclinical, *Clonorchis sinensis* and *Opistorchis verrini* may cause chronic liver infection, pancreatitis, cholangitis and cancer (Choi et al. 2006; Mayer and Fried 2007). *C. sinensis* is widely distributed in East Asia and is endemic to the Red River Delta, whereas *O. viverrini* is present in Laos, Cambodia, Thailand and southern Vietnam.

Approximately, one million people are infected with FZT in Vietnam (Kino et al. 1998). Overall, low to moderate levels of FZT are found within healthy individuals. Epidemiological studies indicate significant geographic variability, associations with culinary habits, and widespread infection of diverse animal species (Table 6). During 2009–2010, an intervention study in 18 fish nurseries introduced snail control by pond draining and treatment of humans and domestic cats. Examination of ~15,000 fish after 9 weeks of intervention indicated moderate success in reducing fish infection rates with FZT (Hedegaard Clausen et al. 2012). Given that human, pig and poultry excreta are commonly used as fish feed, and that snails and fish are fed to poultry, it is likely that multiple vertebrate species play a role in maintaining FZT transmission. There are strong economic and trade incentives to reduce transmission to promote successful development of aquaculture exports.

**Table 6:** Published surveys of foodborne trematode zoonoses (FTZ) in humans and animals in Vietnam.

Citation	Study date	Study location	Sample collections	Sample size	Overall prevalence; additional observations
Phan et al. (2011)	Unknown	Nam Dinh province (Red River Delta)	Faeces of farming household members	180	32.2% FZT egg-positive; 8% did not report eating raw fish; OR = 2.3 for consuming raw fish (vs. no consumption); OR = 3.6 for eating raw fish in restaurants vs. eating raw fish at home
De (2004)	1976–2002	15 provinces all over the country	Healthy individuals, domestic dogs and cats	~30,000	Overall 21% FZT egg-positive for *C. siniensis*/*O. viverrini*. Highest in Nam Dinh (37.5%) and lowest in Thai Binh (0.2%). Prevalence 3 times higher among men. Peak in 40-50 years. Prevalence in dogs (28.6%) and 64.2% in cats. 7/10 species of fresh water fish infected with metacercaria
Kino et al. (1998)	1997	Ninh Binh province (Red River Delta)	Faeces from healthy individuals; tissues of farmed fish	306	13.7% FZT egg-positive for *C. siniensis*; Males higher prevalence than females (23 vs. 1.5%); prevalence increase with age; prevalence of metacercaria in silver carp >56%; prevalence of cercaria among *Melanoides tuberculatus* snails (13%)
Dang et al. (2008)	1999/2000	Ninh Binh province (Red River Delta)	Faeces from healthy individuals	1,115	26.1% FZT egg-positive; males higher prevalence than females; All adult parasites recovered were *C. sinensis;* association between FZT positivity and consumption of raw fish
Olsen et al. (2006)	2004	Nghe An (north-central Vietnam	Faeces of fish farmers	964	0.6% FZT egg-positive for FZT; 0.7% for *Fasciolopsis buski*; infection prevalences of *Ascaris lumbricoides*, *Trichuris trichiura* and hookworm were 34.8, 50.7 and 51.3%, respectively
Trung et al. (2007)	2005	Nam Dinh province (Red River Delta)	Faeces of healthy individuals; positive individuals examined for adult parasites after treatment with praziquantel	615 (33 examined twice)	65% FZT egg-positive; Among treated and re-examined patients: 51% positive with *C. sinensis.* Other species identified were *Haplorchis pumilio* (100%)*; H. taichui* (70%)*; H yokogawai* (3%); *Stellantchasmus falcatus* (6%)*; Fascilopsis buski* (3%)
Chi et al. (2008)	2005	North-central Vietnam	Tissues of tilapia and 6 carp species from 53 fish farms	716	12–61% FZT metacercaria positive species included FZT *H. pumilio*, *H. taichui*, *H. yokogawai*, *Centrocestus formosanus*, *S. falcatus* and *Echinochasmus japonicus*; similar prevalence in nursery and grow-out ponds
Lan-Anh et al. (2009)	2005	Nghe An (north-central Vietnam)	Faeces of terrestrial farm species	35 domestic cats, 80 domestic dogs, and 114 pigs	48% egg FZT positive (cats); 35% (dogs); 14% (pigs)
Nguyen et al. (2007a, b)	2005–2006	Nghe An (north-central Vietnam	Tissues of tilapia and carp fish reared on wastewater-fed ponds	1,200	Overall ~4.8% FTZ metacercaria positive (higher in warmer months). All metacercariae recovered were of the family Heterophyidae. Tilapia and 3 species of carp were infected
Thu et al. (2007)	2005–2006	Mekong Delta	Tissues of catfish and snakehead fish	852	31% FZT metacercaria positive; 10% positive for zoonotic species, including *O. viverrini* (1.9%), *H. pumilio* (2.8%) and *Procerovum* spp. (5.6%)
Anh et al. (2010)	2009	Nam Dinh province (Red River Delta)	Liver tissues from poultry from 60 fish farms	50 (chickens); 50 (ducks)	Identified *Centrocestus formosanus* and *Echnostoma cinetorchis*
De and Le (2011)	2009/2010	Nam Dinh (Red River Delta)	Faeces of healthy individuals; positive individuals examined for adult parasites after treatment with praziquantel	405 (10 examined twice)	32.2% FZT egg-positive; 29.3% in males and 16.0% in females. 385 adult flukes from 10 patients identified: *C. sinensis* (14.6%), Haplorchis taichui (32.3%), *Haplorchis pumilio* (52.08%) and *Centrocestus formosanus* (1.0%)

### Paragonimiasis

Paragonimiasis is a lower respiratory tract infection caused by lung *Paragonimus* flukes. Humans become infected through consumption of infective metacercariae from raw or undercooked crustaceans. Eggs are voided by infected people in sputum or faeces; in the environment, the parasite goes through several stages involving snails and then crayfish or crabs as hosts. Symptoms are sometimes mistaken with chronic tuberculosis (Vijayan 2009). Clinical cases in Vietnam have been documented from mountainous regions, linked to consumption of infected crabs (Table 7). Vietnamese domestic dogs and pigs infected with *Paragonimus* have been reported (Queuche et al. 1997). Species identified from Vietnam include *P.* *heterotremus*, *P.* *vietnamiensis*, *P.* *proliferus* (northern mountainous areas) and *P.* *westermani* (central Vietnam) (Doanh et al. 2007, 2008, 2009). In spite of mass screening, treatment and education programmes, paragonimiasis remains a problem in a limited number of areas of the country.

**Table 7:** *Paragonimus* spp. Studies in Humans in Vietnam.

Citation	Study date	Study location	Sample collections	Sample size	Overall prevalence; additional observations
Queuche et al. (1997)	1993	Lai Chau (northern Vietnam)	Sputum of patients with pulmonary disease; faeces of healthy people	155 patients; 225 healthy; 125 children 8–18 years; 16 domestic dogs; 15 pigs	28% of patients had eggs in sputum; Mean age 11 years; 2 of 155 patients had CNS symptoms; 5% healthy people were egg-positive; associations with consuming freshwater crabs; 5/16 dogs and 2/15 pigs tested positive for adult lung flukes
Vien et al. (1997)	1994/1995	Lai Chau (northern Vietnam)	Sputum of chronic respiratory disease patients	44	2 of 44 with CNS symptoms; 100% of cases egg-positive; most cases had eaten insufficiently roasted crabs
Doanh et al. (2011)	Unknown	Three provinces: Lai Chau, Yen Bai (north) and Quan Tri (central)	Sputum of healthy patients	590	12, 4 and 0% seropositive from Lai Chau, Yen Bain and Quan Tri, respectively; sequences from eggs from sputum of six individuals identified *Paragonimus heterotremus*

### Gnathostomiasis

Gnathostomiasis occurs wherever consumption of raw fish is common. Human infections are acquired by ingestion of advanced third stage larvae (AL3) of *Gnathostoma* spp. present in fish species. Humans are paratenic hosts; the larvae commonly migrate through subcutaneous tissues, visceral organs and the central nervous system. *G. spinigerum* is the most common species in Southeast Asia, usually found in swamp eels (*Monopterus albus*) (Waikagul and Diaz Camacho 2007).

Until 1998 only three cases of *G. spinigerum* had been documented in Vietnam; however, introduction of serological tests since then led to hundreds of cases since. A study indicated that 63.8% had cutaneous and 14.7% had visceral manifestations (Xuan et al. 2004). Severe eye infection due to *G. spinigerum* was reported in the Mekong Delta (Xuan le et al. 2002). Market surveys of eels (*n* = 1,081) in HCMC identified *G. spinigerum* AL3 in 0.11% (Le and Rojekittikhun 2000). Prevalence was higher in wild-caught eels and at the end of the rainy season (Sieu et al. 2009).

### Other FBZ Reported in the Vietnamese Literature

Between 2006 and 2011, 413 human cases and three anthrax deaths were reported in northern Vietnam. All had a history of slaughtering/eating dead ruminants (Tran and Pham 2012). Studies on suspect cases of *Toxocara canis* using serology confirmed 83 visceral and 33 ocular infections (Tran et al. 2001a; Le et al. 2012). A 2004 serological study of 1,201 dairy cattle in HCMC reported negative results for *M. bovis* (ELISA) and *Brucella* spp. (Nguyen et al. 2006).

## Discussion

Our review of 95 publications reveals the highly diverse range of endemic pathogens associated with FBZ in Vietnam. Although a systematic ranking of disease burden associated with FBZ is not possible at this time, the pathogens fall largely into three groups: (1) pathogens that are relatively *more* common as causes of clinical disease in Vietnam than in developed temperate-zone countries; (2) pathogens known to be present in Vietnam that are not responsible for a particularly high disease burden; and (3) FBZ which may be fairly common, but for which the dearth of either research or surveillance data in Vietnam prohibits making any valid assessments of relative burden.

In the interest of maximizing development impacts and pursuing a One Health research agenda, there are clear imperatives to prioritize research on zoonoses within group 1 that also cause significant production losses and incur the highest economic costs to farmers. We suggest that *Streptococcus suis*, *Leptospira*, *Fasciola* and fish-borne trematode infections meet these criteria, and that a better understanding of the transmission ecology of these pathogens within smallholder production systems could readily generate improved control options with benefits to both human and animal health. In contrast, *Campylobacter* and NTS belong to the second category of FBZ, for which the clinical disease does not appear to rank particularly high; although elsewhere in the world *Campylobacter* and NTS are dominant causes of foodborne diarrhoea, and are the focus of intense multinational control efforts. The influence of human–animal contact rates and human population immunity to *Campylobacter* and NTS merits further research, since future changing patterns of exposure may drive a shift in the age-related incidence of infection. Unfortunately, the majority of pathogens fall within category 3, for which data sources are entirely inadequate to estimate burden.

Potential impacts of ongoing urbanization and economic development on FBZ in Vietnam are summarized in Table 8, alongside suggested areas for further research and improved surveillance. Surveillance of FBZ remains one of the weakest aspects of the health systems in Vietnam. In most cases, hospitals do not carry out routine diagnosis of most bacterial and parasitic FBZ. Serious diseases such as leptospirosisa and toxoplasmosis are often not adequately diagnosed and reported.

**Table 8:** Summary of main challenges and suggested priority research areas on FBZ in Vietnam.

Foodborne pathogen	Data on prevalence/ incidence in humans	Data on animal reservoir	Challenges for Vietnam	Suggested areas of research
Non-typhoidal *Salmonella* (NTS) infection	Responsible for ~0–7% of diarrhoea in <5 year children; limited data on serovar distribution in humans. High levels of NTS carriage among adults; some evidence for person-to-person transmission among children	High prevalence and variability of serovars in poultry, pigs, fish/seafood and meat products	Meeting export targets of meat products will require improved control of NTS in fish, pigs and poultry	Attribution studies in humans; impact of urbanization and backyard farming on human immunity; Antimicrobial resistance; NTS diversity within backyard versus industrialized production systems
*Campylobacter* spp. infection	Responsible for ~4–40% of diarrhoeal cases in <5 year children (Red River Delta)	Very high prevalence in chicken carcasses and meat products	High levels of multi-resistance including ciprofloxacin resistance	Risk factors and attribution studies among clinical cases; prevalence and genetic diversity in backyard versus industrialized production systems, and along processing/ retailing market chains; impact of urbanization and backyard farming on human immunity
*Streptococcus suis* infection	Most common cause of adult bacterial meningitis; majority of cases caused by *S. suis* serotype 2; approximately 5–43 confirmed cases per year in Vietnam	High (>40%) carriage in upper respiratory tract (tonsils) of market weight swine; predominance of *S.* *suis* 2; epidemiological interactions with viral infections (e.g. PRRSv)	Improved control over illegal marketing of ill pigs; hygiene and health quality standards in slaughter/processing facilities	Estimation of burden of disease using combined indicators for human morbidity/mortality and economic losses to swine sector; risk factors for pig colonization; development of porcine vaccines and novel diagnostic tools for herd management and risk mitigation
Listeriosis	Three clinical case reported from north Vietnam with meningitis in 2008/2009	No data	Possible increased incidence in coming years, due to greater consumption of packed food items including soft cheeses, meat and fish	Consumer perceptions of risk, health and safety in relation to highly processed foods; enhanced surveillance among high risk groups; investigation in food processing plants
Toxoplasmosis	No published data on human clinical cases. Seroprevalence in humans ranging between 1.1 and 6.4%. Higher (7.7–11%) among pregnant women and drug users in some	Very high prevalence in domestic dogs and pigs (>50%); lower in cattle and buffalo (3–10%)	Unknown risks due to poor understanding of principle zoonotic reservoir	Enhanced surveillance among pregnant women and neonates to estimate burden of disease; prevalence of oocysts in cats, dogs, dog meat and treated and untreated wastewater
Cryptosporidiosis	No published data on human clinical cases. At least two studies of paediatric diarrhoea failed to identify *Cryptosporidium*	High prevalence of *C. parvum* and *C andersoni* in cattle; *Cryptosporidirum* spp. oocysts found in pigs and farmed fish but not speciated	Risks associated with uncontrolled urbanization, peri-urban agriculture, waste water treatment and climate change	Etiological and syndromic studies of enteric disease in humans and animals; development of informal networks for reporting and investigating suspect foodborne outbreaks
Giardiasis	No published data on human clinical cases. 4% carriage of *G. lamblia* among healthy subjects in north Vietnam	50% of calves near Hanoi found positive by feacal microscopy; however, dominant species may be non-zoonotic *G. duodenalis*	Risks associated with uncontrolled urbanization, peri-urban agriculture, waste water treatment and climate change	Etiological and syndromic studies of enteric disease; species diversity in farm animals and farmed ’wild’ exotic species; risk factor studies; detection of oocysts in vegetables, treated and untreated wastewater
Taeniasis/ cysticercosis	In the late 1990s, approximately 100–150 cases/year with cerebral cysticercosis in northern Vietnam. Human surveys (2003/2004) using stool egg counts suggest low level prevalence of (0.2–7.2%). Likely to be circumscribed to certain areas in Vietnam	Multiple species identified from pigs and domestic dogs, including *T. solium*, *T saginata asiatica*	Probable future reductions in prevalence/incidence due to changes in swine production	Seroepidemiological and clinical studies. Identify host species of *T. s. asiatica*; studies investigating prevalence of *Taenia* eggs in the environment
Fascioliasis	>1,000 patients/year reported in central provinces, especially Quang Nai; seroprevalence ~8% in some areas; diagnostic case reports increasing	Hyper-endemic in ruminants of central provinces (>70% in adult cattle); high level species diversity; hybrid species identified (*F. gigantica* and *F. hepatica*)	Risks associated with changes in forage production for beef and dairy cattle	Detection of metacercariae in leaf vegetables; ecologic determinants of disease transmission; risk assessment; development of novel indicators to estimate combined disease burden in humans and animals
Leptospirosis	Highly seroprevalence in southern Vietnam suggesting endemicity. Responsible for 2–8% cases of acute jaundice. Main serovars identified Seramanga and Bataviae	Hyper-endemic in pigs in the Mekong Delta	Very common in kidneys in fattening pigs. Main serovars Bratislava, Iterohaemorrhagiae, Automnalis and Pomona	Estimate burden of infection by targeting patients with suspect hepatic and haemorrhagic syndromes. Investigate main reservoirs of infection including rats, pigs, dogs and cattle
Trichinellosis	Decreasing incidence in recent years; small outbreaks in northwest	Seroprevalence in swine ~14–20% in some areas	Probable future reductions in prevalence/incidence due to changes in swine production	Role of rodents in transmission; risks associated with specific culinary practice
Fishborne zoonotic trematode (FZT) infection	High rates of asymptomatic carriage in humans living in Red River Delta provinces (>75%)	High species diversity including both pathogenic and non-pathogenic flukes of multiple genera	Risks associated with expansion of aquaculture industry, waste water treatment and climate change	Enhanced surveillance to estimate disease burden; detection of FZT in fish; risk assessment; intervention studies; ecologic determinants of disease transmission

The pace of industrialization of Vietnam’s farming systems varies by sector and region. The trend is towards increasing farm sizes with higher stocking densities and modern management (all-in all-out systems, synchronized breeding, etc.). In the last decade, central decisions made at the Ministry of Agriculture and Rural Development and the Department of Livestock Production to promote restructuring of the poultry sector was viewed as a way to improve control of highly pathogenic avian influenza (HPAI). Although consolidation undoubtedly provides many more opportunities for increased biosecurity at the farm level, it may also increase vulnerabilities to dissemination of pathogens across the food chain. Changes in pathogen exposure, increased stress and breed and management factors may alter herd/flock immunity and pathogen population dynamics. The risk of pathogen emergence in modern versus traditional production systems has received some attention, but largely in relation to viruses (Drew 2011; Graham et al. 2008). It remains to be seen whether knowledge gained on drivers of viral emergence can be generalized to bacterial and parasitic FBZ.

In spite of government efforts to promote consolidation, smallholder mixed crop–livestock production remains dominant in Vietnam. Use of animal/human excreta and feed leftovers is common, especially within the ‘VAC system’ (Vuon = garden, Ao = pond and Chuong = pig pen) (Pham Duc et al. 2011). Such integrated systems provide efficient nutrient recycling, but also may promote transmission of parasites whose life cycles involve invertebrates. VAC systems are now less common in Vietnam than a few decades ago, due to alternatives for use of animal excreta (i.e. biogas) as well as increasing constraints on land use and increased land costs. Government programmes and development projects aimed at improving sanitation have resulted in safer human waste disposal. Where human excreta are used as fertilizer, a minimum of 6-month retention period is recommended to ensure pathogen inactivation. The level of compliance with this norm is not known, although some data suggests good adherence (Phuc et al. 2006). VAC systems are of course particularly vulnerable to fish-borne trematode infections, whereas industrial aquaculture operations provide increased investments in infrastructure for both quality and safety control, through the use of commercial laboratories for pathogen screening and chemical pest control of invertebrates. In the swine sector, investments in housing and improved nutrition are expected to reduce the burden of parasitic diseases such as taeniasis/cysticercosis and trichinellosis. Intensified bovine and dairy production may increase the risk of introducing cattle-associated FBZ such as bovine tuberculosis and brucellosis. Finally, for target organisms that are particularly associated with processed animal foods, such as listeriosis, increased consumption of processed food items such as soft cheeses, sausages and pates may result in increased incidence unless production of these commodities is adequately regulated.

In Vietnam, per capita ingestion of animal protein has steadily increased over the last few years (Thang and Popkin 2004) and in urban areas, the consumption of chilled, frozen and processed meat is rapidly increasing (Anon. 2011). Modern retail outlets (supermarkets, convenience stores, etc.) now account for >15% of total food distribution (Cadilhon et al. 2006), and fast-food restaurants are rapidly proliferating. Consumption of wild-animal meat has also been increasing among wealthy sectors of the population; these ‘exotic’ products pose novel and unforeseen food safety risks (Drury 2011).

In the past, regulation of food safety in Vietnam has been hampered by highly decentralized authority for monitoring value chains. A Food Safety Law (No. 55/QH12/2010) seeks to impact quality control of slaughter and processing facilities within food distribution networks, in part through clarifying new standards and regulatory policies. Examples include the development of certification systems for good food production and slaughtering practices, increase traceability and strengthening of penalties for marketing uncertified animals. Better control of food chains is likely to improve control of diseases associated with unregulated marketing (i.e. *S. suis*). In addition, measures such as zoning regulations on the proximity of production units close to open waterways or urban centres have been introduced. Although the impetus for many of these reforms is driven by the threat of avian influenza pandemics, the measures will likely have an impact both on disease transmission and cultural practices. Efforts to expand export markets of agricultural commodities are also providing an incentive to improve quality controls and laboratory testing; these developments are likely to be driven by the private sector and will target organisms such as NTS to meet international regulatory standards.

In summary, the rapid intensification of animal food production systems and urbanization in Vietnam will undoubtedly change the landscape of food safety risks, introducing both new opportunities for control and prevention, as well as new vulnerabilities for the spread of disease. Within this context, the key for understanding and monitoring changes will be a strengthened infrastructure for surveillance, both of human clinical disease and within the veterinary community.
